# VT-3DCapsNet: Visual tempos 3D-Capsule network for video-based facial expression recognition

**DOI:** 10.1371/journal.pone.0307446

**Published:** 2024-08-23

**Authors:** Zhuan Li, Jin Liu, Hengyang Wang, Xiliang Zhang, Zhongdai Wu, Bing Han

**Affiliations:** 1 College of Information Engineering, Shanghai Maritime University, Shanghai, China; 2 National Engineering Research Center of Ship Transportation Control Systems, Shanghai Ship and Shipping Research Institute, Shanghai, China; Zhejiang Normal University, CHINA

## Abstract

Facial expression recognition(FER) is a hot topic in computer vision, especially as deep learning based methods are gaining traction in this field. However, traditional convolutional neural networks (CNN) ignore the relative position relationship of key facial features (mouth, eyebrows, eyes, etc.) due to changes of facial expressions in real-world environments such as rotation, displacement or partial occlusion. In addition, most of the works in the literature do not take visual tempos into account when recognizing facial expressions that possess higher similarities. To address these issues, we propose a visual tempos 3D-CapsNet framework(VT-3DCapsNet). First, we propose 3D-CapsNet model for emotion recognition, in which we introduced improved 3D-ResNet architecture that integrated with AU-perceived attention module to enhance the ability of feature representation of capsule network, through expressing deeper hierarchical spatiotemporal features and extracting latent information (position, size, orientation) in key facial areas. Furthermore, we propose the temporal pyramid network(TPN)-based expression recognition module(TPN-ERM), which can learn high-level facial motion features from video frames to model differences in visual tempos, further improving the recognition accuracy of 3D-CapsNet. Extensive experiments are conducted on extended Kohn-Kanada (CK+) database and Acted Facial Expression in Wild (AFEW) database. The results demonstrate competitive performance of our approach compared with other state-of-the-art methods.

## 1 Introduction

Facial expression recognition has been widely used in the fields of mental health, virtual reality, synthetic animation, intelligent monitoring, and intelligent robots. A FER system [1] is mainly composed of three stages: face detection, facial feature extraction, and expression recognition. Generally, Face detection detects whether there is a face in the given image area based on unique facial characteristics and then locates the facial coordinate and segments the face from the image. Facial feature extraction is only performed on the facial area, which is the most important stage of FER. After the facial expression features are obtained, the facial expressions are classified through using the neural network based method. The robustness and completeness of the extracted features will decisively influence the final recognition result.

With the development of convolutional neural network in many fields [2–5], substantial breakthroughs have been made in facial expression recognition. Zhang et al. [6]. proposed a Part-based Hierarchical Bidirectional Recurrent Neural Network (PHRNN) to analyze the facial expression information of temporal sequences, which employed a multi-signal convolutional neural network (MSCNN) to extract spatial features from still frames to complement the still appearance information. Fan et al. [7] proposed MRE-CNN framework for FER, which aimed to enhance the learning power of CNN models by capturing both the global and local features. Li et al. [8] put forward a new DLP-CNN method that enhanced the discriminative power of deep features by preserving the locality closeness while maximizing the inter-class scatter. However, these methods for FER only focus on local or global feature representation, and they neglect relationship (relative position relationship, scale, feature direction, etc.) among local facial features [9]. There are several important studies as follows:

The proposal of the capsule network [10] can well tackle the above problems. The capsule network uses neuron vectors to learn the pose information for targets and uses dynamic routing mechanism to transfer the capsule information between layers. This network has achieved the higher accuracy rate and strong robustness in digital recognition. However, the traditional capsule network only uses one layer of convolution for spatial feature extraction, which limits its performance [11]. Therefore, in the 3D-CapsNet model proposed in this paper, the 3D convolutional architecture is used in the feature extraction stage to extract spatiotemporal features, and then extraxted features are further encoded by dynamic routing mechanism. In addition, the visual tempos of facial expressions [12] can be employed to improve the recognition effect. Specifically, different facial expressions usually hold different visual tempos. However, In some cases there are larger similarities in visual appearance of different expressions(e.g. Fear and Surprise) and the key to distinguish them is their visual tempos.

To this end, we utilize improved deep 3-dimensional convolutional neural network model(3D-ResNet) to replace the traditional convolutional layer of capsule networks, and we named the improved capsule network that applyed improved 3D-ResNet architecture 3D-CapsNet. Specifically, the improved 3D-ResNet architecture employs 3D convolution kernels to learn spatiotemporal features in the residual block part. Meanwhile, we incorporate AU-perceived attention module into the 3D residual block to better focus on the key facial area. Then a non-local attention block is used to capture long-range dependencies on spatial and temporal dimension. Next we exploit dynamic routing algorithms of CapsNet to calculate the weight allocation between different capsules to capture the hierarchical structure of features and better handle complex feature relationships.

Given that 3D-CapsNet focuses on improving the ability of feature representation, the ability of modeling the dynamic visual tempos of facial expressions is insufficient. Therefore, we propose TPN-based expression recognition module (TPN-ERM) to further improve our 3D-CapsNet model and we named the improved 3D-CapsNet architecture that integrated with TPN-ERM VT-3DCapsNet. Overall, our main contributions can be summarized as follows:

1. First, we propose 3D-CapsNet model for emotion recognition, in which we introduced improved 3D-ResNet architecture that integrated with AU-perceived attention module to enhance the ability of feature representation of capsule network through extracting deeper hierarchical spatiotemporal features and extracting latent information (position, size, orientation) in key facial areas.
2. Second, we propose the temporal pyramid network(TPN)-based expression recognition module(TPN-ERM), which can learn high-level facial motion features from video frames to model differences in visual tempos, further improving the recognition accuracy of 3D-CapsNet.
3. Finally, extensive experiments are conducted on the CK+ and AFEW datasets, the results demonstrate that our method significantly outperforms other state-of-the-art methods.

The remaining of this article is organized as follows. The related work is presented in Section 2, while the 3D-CapsNet model and TPN-based expression recognition module are discussed in Section 3. Experimental results and discussion are shown in section 4, and conclusion is drawn in Section 5.

## 2 Related work

### 2.1 Traditional hand-crafted feature extraction for FER

Most of the traditional facial expression feature extraction algorithms used artificially designed features or shallow learning methods. Local Binary Pattern (LBP) is one of the finest manual feature extraction techniques. Niu et al. [13] offered an approach based on a combination of LBP features and an enhanced ORB, which effectively solved the problem of overlapping and redundant feature points in the feature extraction process and produced decent results on multiple experimental controlled datasets. Xiang et al. [14] proposes a novel illumination insensitive feature descriptor by integrating the center-symmetric local binary pattern (CS-LBP) into a common feature description framework. However, these approaches, due to their low semantic intensity, fail to perform accurate recognition in more complex real-world scenarios. To tackle this problem, Liao et al. [15] introduced RCL-Net that is based on ResNet-CBAM residual attention branch and the local binary feature (LBP) extraction branch (RCL-Net), which aimed to emphasize the local detail feature information of facial expressions and extract texture feature information. However, these manual feature extraction approaches have a high workload, lengthy stages and significant restrictions in terms of practical applications, and these methods depend heavily on researcher’s experience and their performance is strongly affected by image quality variations.

### 2.2 Deep learning based methods for FER

Recently, the methods based on deep learning have been widely used for FER and achieve state-of-the-art performance. But most CNN-based models fail to learn long-range inductive biases between different facial regions in most neural layers, which limits the performance of the models. To address this problem, Huang et al. [16] introduced a novel FER framework with two attention mechanisms for CNN-based models. In particular, a visual transformer attention mechanism is used to learn high-level semantic representation. Wu et al. [17] proposed a novel cross-hierarchy contrast (CHC) framework FER-CHC to utilize these crucial features in improving the performance of CNN-based models for FER through employing a contrastive learning mechanism. Specifically, the CHC captures common and differential features from different facial expressions with a cross-hierarchy contrast mechanism, which can regularize the feature learning of the backbone network and enhance global representations of facial expressions. Recent methods based on Vision Transformer (ViT) [16, 18] have been introduced to improve the performance of CNN-based models. However, ViT-based approaches are vulnerable to facial regions unrelated to expressions and may learn redundant correlation representations due to their self-attention mechanism. To address these issues, Fan et al. [19] proposed a novel graph-based model called Face2Nodes, which can flexibly learn the graph representations of facial expressions without requiring additional auxiliary facial information such as landmarks. In addition, Zhou et al. [20] realized that existing models failed to capture the crucial complementary gains between face and context information in video clips. And they presented a novel cross-attention and hybrid feature weighting network to achieve accurate emotion recognition from large-scale video clips through fully exploiting the complementary information between face and context features.

Kensho et al. [21] proposed a 3D CNNs based on ResNets toward a better action representation and described the training procedure of 3D ResNets in details. Teng et al. [22] proposed a new network called Typical Facial Expression Network (TFEN) to extract temporal features and the spatial structure of facial expressions in an integrated manner, which uses two deep two-dimensional (2D) convolutional neural networks (CNNs) to extract facial and expression features from input video. A facial feature decoupler decouples facial features from expression features to minimize the influence from inter-subject face variations. These networks combine with a 3D CNN and form a spatial-temporal learning network to jointly explore the spatial-temporal features in a video. But 3D convolutions usually employ structures with fixed temporal depth that decreases the potential to extract discriminative representations. Based on these, Melo et al. [23] proposed a novel deep learning architecture called the Maximization and Differentiation Network (MDN) to effectively represent facial expression variations that are relevant for depression assessment. Khanna et al. [24] combines two commonly used deep 3-dimensional Convolutional Neural Networks (3D CNN) models with slight modifications. Initially, the 3D ResNet model extracted feature vectors from video frame sequences, then these feature vectors are fed to the 3D DenseNet model’s blocks, which are then used to classify the predicted emotion.

The convolution neural network does not consider the spatial and interlayer features of the target. The use of dynamic routing in capsule networks [10] can well address the above problems. However, the traditional capsule neural network does not extract features sufficiently before the dynamic routing between the capsules. To achieve better feature representation, Shu et al. [25] presented RES-CapsNet to investigate the recognition of micro-expression, which proposed an improved capsule network that used Res2Net as the backbone to extract multi-level and multi-scale characteristics. low-quality 3D face recognition(FR) with missing facial features still suffers from insufficient discriminative feature extraction for visible face regions. Zhao et al. [26] proposed a dual-stream multi-scale fusion network (DSNet) for low-quality 3D FR. Which introduced a capsule network as the second stream to enhance the expression of 3D facial spatial position information, thereby further improving the performance of low-quality 3D FR with missing facial features. Ye et al. [27] designed SCapsNet for FER, which used a shallow small convolution kernel to reduce the network parameters of the capsule network and optimize the quantity and quality of capsule of the capsule network to improve the calculation efficiency.

Feature pyramid is a widely-used method to learn multi-scale feature representation for detecting objects of various scales. FPN [28] exploited the inherent multi-scale pyramidal hierarchy of deep convolutional networks to construct feature pyramids with marginal extra cost. Liu et al. [29] proposed PANet which added a bottom-up path to enhance the entire feature hierarchy with accurate localization signals in lower layers. However, FPN and PANet cannot handle the information about temporal series well, and they are designed in complex and multi branch manner. On the other hands, visual tempo characterizes the dynamics and the temporal scale of an action. Therefore, modeling such visual tempos of different actions will facilitate their recognition. Yang et al. [12] proposed a temporal pyramid network (TPN) for modeling the visual tempo. The extraction of features and fusion of features form feature hierarchy structure for the backbone so that they can capture action instances at various tempos. Yang et al. [30] proposed a CNN-based framework called Pyramidal Spatio-Temporal Network (PSTNet), which employed Spatial encoding for spatial representation of external factors, while prior pyramid enhances feature dependence of spatial scale distances and temporal spans, then post pyramid is proposed to fuse the heterogeneous spatio-temporal features of multiple scales. To enrich temporal information of the inputs, a Multiple Frame Rate Module (MFM) is proposed to mix different frame rates at a fine-grained pixel-wise level. Chen et al. [31] introduced a Multiple Frame Rate Module (MFM) which mixed different frame rates at a fine-grained pixel-wise level to enrich temporal information of the inputs. But these methods remain computationally expensive when dealing with the dynamic visual tempos of action instances at the input frame level. In this work, we improve the ways of feature fusion and introduce adaptive weighted fusion to better learn parameters in the feature fusion stage to tackle this problem.

To sum up, the performance of FER system can still be greatly improved. In the existing works, failing to make full use of spatiotemporal features limits the performance of the model, and visual tempos of facial expressions are rarely used in FER. As a result, we propose a fine-grained method that uses 3D-CapsNet to fully utilize and encode the feature presentation in the input video. We also propose TPN-ERM, which employs visual tempos of different facial expressions to further improve the performance of 3D-CapsNet.

## 3 Our methods

**Fig 1:**
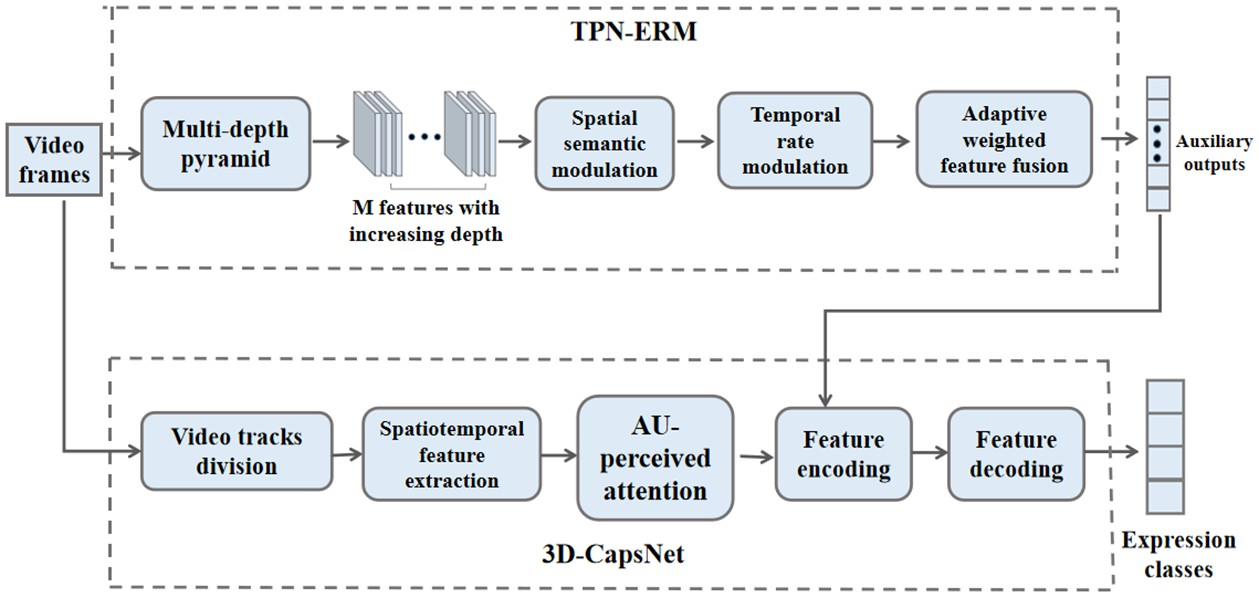
An illustration of our visual tempos 3D-capsule network framework(VT-3DCapsNet). 3D-CapsNet is made to learn a feature vector close to high-level motion feature via connecting auxiliary outputs to the PrimaryCaps layer for feature encoding.

An overview of the proposed approach is depicted in Fig 1. The input of the 3D-CapsNet is limited to a small number of contiguous video frames owing to the increased trainable parameters as the size of input window (the time dimension of the convolution) increases. On the other hand, many facial actions of expressions are extended in various frames, it is necessary to encode facial motion information in the 3D-CapsNet model. Toward this end, we propose to use improved TPN-ERM to calculate facial motion features representing expressions from a considerable number of video frames and use these features as auxiliary outputs to regularize 3D-CapsNet model as shown in Fig 1. Particularly, we generate a feature vector encoding long-term facial motion information beyond the information contained in the input frames to the 3D-CapsNet for each trained expression. 3D-CapsNet is made to learn a feature vector close to this feature, which is accomplished via connecting many auxiliary output units to the PrimaryCaps layer in 3D-CapsNet. This will enable 3D-CapsNet to learn high-level expression features.

In the following, we describe our proposed 3D-CapsNet model which is used to better learn spatiotemporal feature. Then, we describe TPN-based expression recognition module (TPN-ERM) that models the variances in visual tempos to further optimize the performance of 3D-CapsNet model.

### 3.1 The 3D-CapsNet model

**Fig 2:**
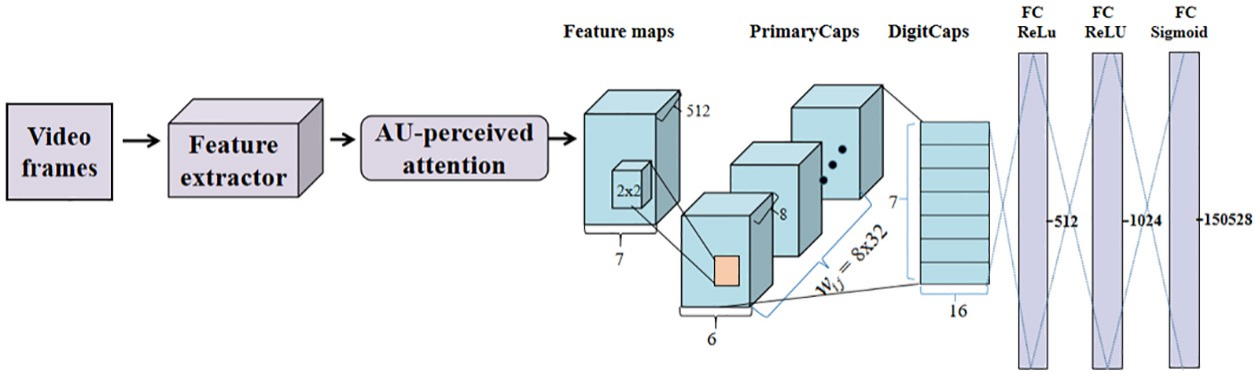
The overall architecture of 3D-CapsNet. (1) The improved 3D-ResNet servers as a feature extractor to better learn spatiotemporal features, and the AU-perceived attention mechanism is introduced to perceive the specific AU changes related to facial expression and focus on effective feature information of key facial areas. Thus enhancing model’s ability of feature representation. (2) The capsule network module encodes the enhanced feature mapping through dynamic routing mechanism, decodes it through three fully connected layers, and implements the final expression classification through the squeeze function.

The overall architecture of 3D-CapsNet is shown in Fig 2. The improved 3D-ResNet extracts deep spatiotemporal features from video tracks (which can be regarded as a video clip). And the encoding and decoding abilities of the dynamic routing mechanism in the capsule network can obtain features represented by vectors, thus improving the accuracy of facial expression recognition. Each module of 3D-CapsNet will be described in detail in the following sections.

#### 3.1.1 Spatiotemporal features extraction

**Fig 3:**
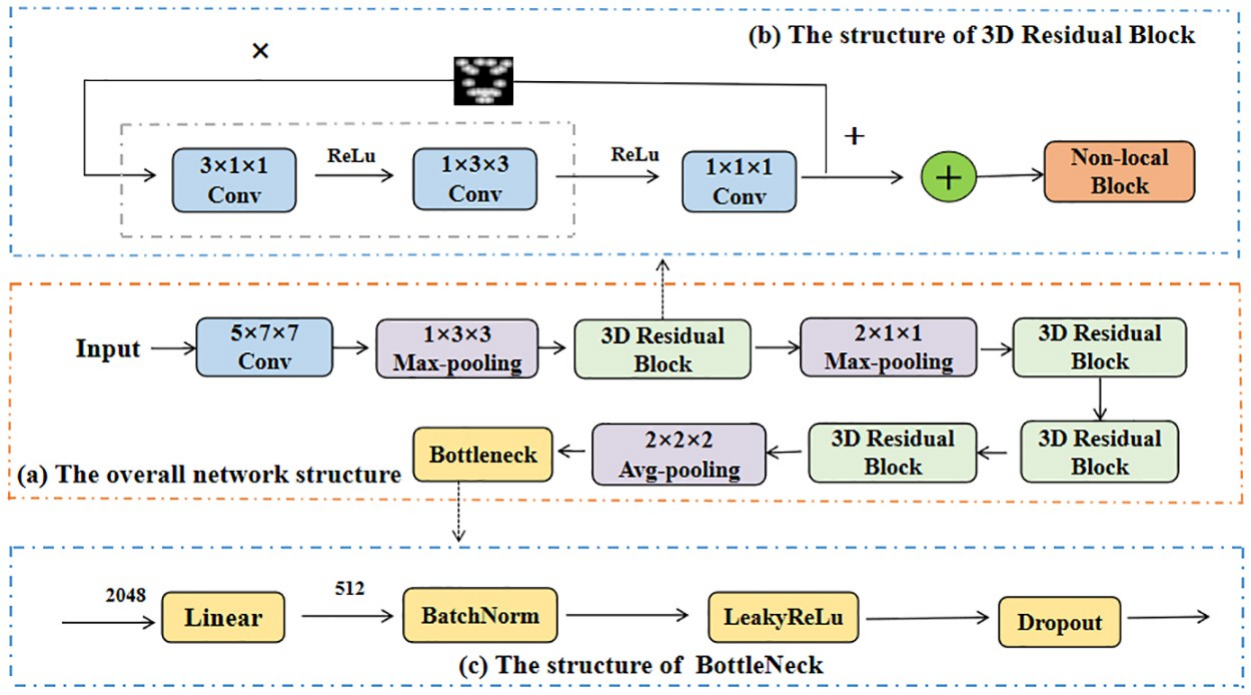
The overall structure of improved 3D-ResNet.

The whole system could be divided into two important parts: extracting spatiotemporal features from video tracks through improved 3D ResNet, integrating spatiotemporal features by the non-local blocks, and learning the features in key facial areas through the AU perception enhancement module. The video clip is first divided into continuous non-overlapping small segments, and each small segment contains *N* frames. Assuming that each fragment is represented as:

$$\begin{array}{c} c_{k}={\left\{x_{t}|x_{t}\in R^{H\times W}\right\}}_{t=1}^{N} \end{array}$$ (1)

where *N* is the length, *H* and *W* are the height and width of the image respectively. Specifically, our network structure is shown in Fig 3, in which (a) is the overall improved 3D ResNet structure, (b) is the residual block structure of the 3D-RseNet we used, and (c) shows the bottleneck block after average pooling to speed up training and improve performance.

We adopt 3D convolution kernels of 3D ResNet-50 architecture to extract spatiotemporal features. However, 3D-ConvNet is hard to optimize because of the large number of parameters. To tackle this problem, we added an extra time dimension to all 2D ResNet-50 convolution filters. For example, a 2D *k* × *k* kernel can be exaggerated to a 3D *t* × *k* × *k* kernel that spans *t* frames. Specifically, we use a 1D convolution layer which is purely to learn temporal sequence features and a 2D convolution layer to learn the spatial features in the residual block. As shown in the blue dashed box in Fig 3(b). Meanwhile, we have also incorporated AU-perceived enhancement module into the 3D residual block to better focus on the key parts of the facial area. Finally, our network also contains a non-local attention block [32] to capture long-range dependencies on spatial and temporal dimension.

#### 3.1.2 The AU-perceived attention module

Since the face in video is an image containing structured targets(facial organs), the contribution of facial active areas will be more prominent. Facial expression recognition needs to pay more attention to the facial key areas. We refer to method proposed by Wei Li [33] and show specific implementation algorithm of attention mapping in Table 1.

**Table 1:** The algorithm of attention map generation.

**Algorithm 1 Attention map generation algorithm.**
**Input:** The path of face image(path); Key area of the face(keyArea);
**Output:** Coordinates of the bounding box(bbc); the attention map(am).
1: image ← Image.open(path)
2: img_array ← np.array(image)
3: landmark_point ← ObtainLandmark(keyArea, img_array)
4: au_point ← ComputeAuCenter(landmark_point, img_array)
5: dm ← ManhattanDistance(au_point)
6: wa ← 1 − 0.095 * dm
7: bbc, am ← GetBoundingBox(wa, au_point, img_array)
8: return bbc, am

First, 68 landmarks based on the key area of the face are obtained, and then the AU center is gained by moving the zoom distance or using the existing landmarks. The rules for defining AU centers also refer to Wei Li [33]. Finally, we build the attention map for AUs based on key facial landmarks.

After that, we adjust the attention map to the 100 x 100 pixels to ensure that the attention map of each image is uniform. For each AU center, 7 pixels near the center are regarded as AU area, so the size of each AU area is 15 x 15 pixels. Higher weight is assigned to the closer points to the AU center. The relationship follows the equation:

$$\begin{array}{c} w_{\alpha }=1-0.095d_{m},\;d_{m}=\left|x_{1}-x_{2}\right|+\left|y_{1}-y_{2}\right| \end{array}$$ (2)

where *d*_*m*_ is the Manhattan distance to the AU center. Suppose the coordinates of the two points are (*x*_1_, *y*_1_) and (*x*_2_, *y*_2_) respectively. The Manhattan distance represents the sum of the absolute axis distances of the two points on the standard coordinate system. We use a relatively large local area to cover the key areas of AU so that the generation method of attention mapping can have a certain degree of fault tolerance to landmark shift.

**Fig 4:**
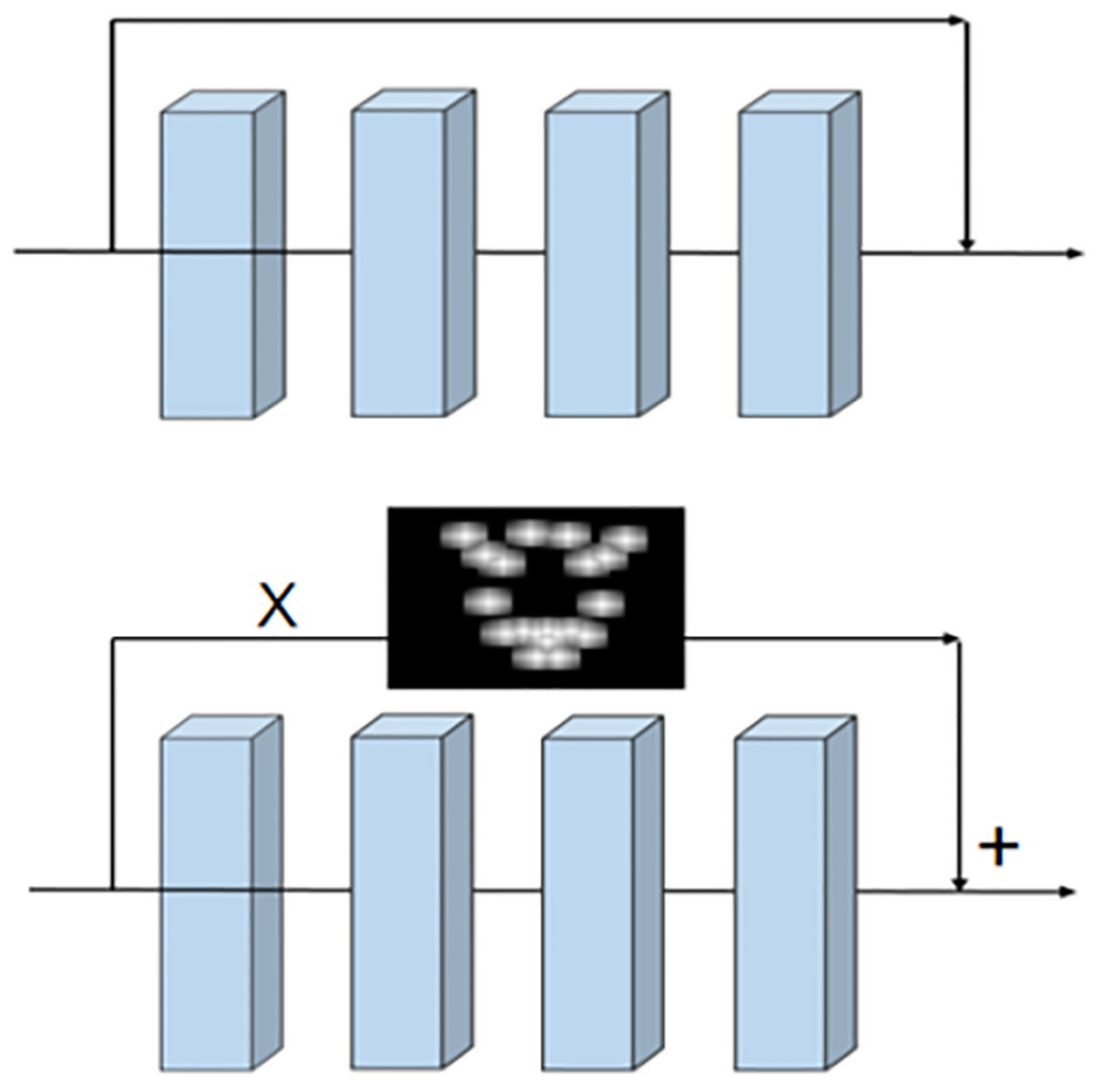
Skipping layer connection in ResNet and application of attention map in our model.

However, applying the attention layer directly will discard a lot of information which are not included in the attention layer. We use the skipping layer of the residual network to combine the attention enhancement area with other areas so as to address the problem of losing information in non-critical areas. Fig 4 shows the skipping layer connection in residual network and attention mapping in our model. The skipping layer structure combines the lower level spatial features in the convolution layer with the higher-level semantic features to form richer feature representation of the input image.

As shown in Fig 3, the specific operation is to embed the generated attention map in the convolution operation of the first 3D residual block, and set the attention map as a parallel stream separately. After relevant feature map is generated through the foregoing maximum pooling, we will multiply the feature map by the attention map. After the convolution in the 3D residual block is completed, the parallel attention feature generation map and the convolution result will be added element by element for fusion to input the pooling layer of next stage.

#### 3.1.3 Feature encoding and decoding

**Fig 5:**
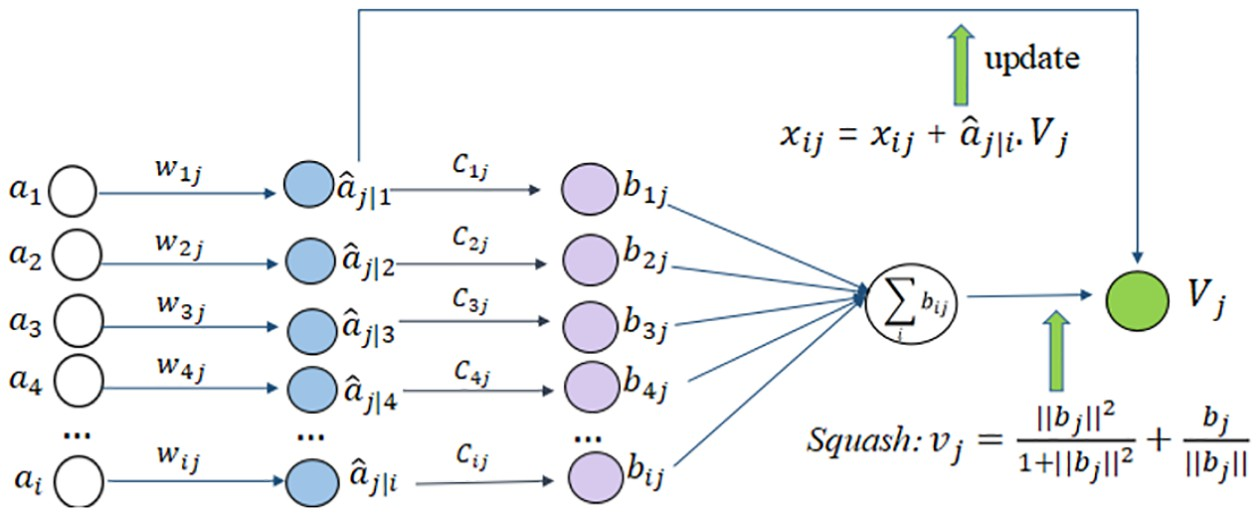
Dynamic routing process.

The capsule network (CapsNet) uses neuron vectors instead of traditional neuron nodes. These neuron vectors can characterize in an image, such as the position, size, and orientation of an object. Therefore, the features of the image can be comprehensively learned. We use the length of the output vector to express the probability of the existence of the entity, and the direction of the vector represents the instantiation parameter. A nonlinear squeeze function called squash is used to compress the capsule vector length to a value between 0 and 1. The longer the length is, the higher probability of the entity appearing in the input. To prevent the loss function from failing to converge when the length of the vector is 0, a minimal value *ε*(10^−7^) is added to ‖*b*‖ to ensure that training proceeds normally, as shown in Eq 4. The formula of the “squashing” function is as follows:

$$\begin{array}{c} \nu_{j}=\frac{{\left\|b_{j}\right\|}^{2}}{1+{\left\|b_{j}\right\|}^{2}}+\frac{b_{j}}{\left\|b_{j}\right\|} \end{array}$$ (3)

$$\begin{array}{c} \begin{array}{c} \left\|b\right\|=\sqrt{\sum_{i}b_{i}^{2}+\varepsilon },\\ b_{j}=\sum_{i}c_{ij}w_{ij}\alpha_{i},\\ \;{\hat{\alpha }}_{j|i}=w_{ij}\alpha_{i},\\ \;c_{ij}=\frac{exp(x_{ij})}{\sum_{k}exp(x_{ik})} \end{array} \end{array}$$ (4)

here *ν*_*j*_ is the output vector of the output layer, and *b*_*j*_ is the input vector of the output layer, which is a weighted sum of the prediction vectors ${\hat{\alpha }}_{j|i}$. These prediction vectors are obtained by multiplying the output *α*_*i*_ of the fully connected capsule layer and a pose matrix *w*_*ik*_, as shown in Eq 4. *c*_*ij*_ is the coupling coefficient determined by the iterative dynamic routing process. This variable is used to measure the consistency between the fully connected layer capsules and the output layer capsules. The initial value of *x*_*ij*_ is set to 0, thereby ensuring that the prior probabilities of the information transmitted by the low-level capsules to the high-level capsules are equal. The coupling coefficients are iteratively determined from the initial values, and the dynamic routing process is shown in Fig 5.

The capsule network architecture and specific network parameters are shown in Fig 2. The 512 x 7 x 7 feature map obtained through the AU attention-constrained dual enhancement network are sent to the PrimaryCaps layer, and a 2 x 2 convolution kernel with a step length of 1 is used to obtain a 256 x 6 x 6 feature map, which is adjusted to 32 8-dimensional feature vectors, the feature map size is 6 x 6. Between the PrimaryCaps layer and the DigitCaps layer, each capsule receives input from all capsules in the previous layer, and the network executes a dynamic routing consensus algorithm. The output of the DigitCaps layer is a 16 x 7 dimensional vector, where 16 represents the vector dimension and 7 represents the number of expression category.

For the capsules in PrimaryCaps layer, the activated capsules encode position information. After passing through the DigitCaps layer, the activated capsule transfers the spatial position information of the image to the predicted probability output of the vector. The transition from position information encoding to probability encoding indicates that as the level of the capsules increases, the dimensionality of the capsules should increase. From the perspective of the proposed architecture, the dimension of capsules in the PrimaryCaps layer is an 8-dimensional vector, while the dimension of capsule in the DigitCaps layer is 16 dimensions. The high-level capsules possess more degrees of freedom and can represent more complex entities.

We have three fully connected layers for image reconstruction after the DigitCaps layer, which is the decoding stage of capsule network. The real label is used as the reconstruction target in the training process. The loss function of image reconstruction is constructed by calculating the Euclidean distance between the output pixels of the Sigmoid layer and the pixels of the original image. The coupling coefficient *c*_*ij*_ is updated through dynamic routing, while the update operation of other convolution parameters and the weight matrix in the capsule is completed through the loss function. Our loss function contains margin loss *L*_*c*_ and reconstruction loss *L*_*r*_, and we add a scaling factor *ρ* to reconstruction the loss to make the margin loss dominate the training process and not be dominated by the reconstruction loss *L*_*r*_. The formula of each loss function is as follows.

$$\begin{array}{c} L_{c}=T_{c}max{\left(0,m^{+}-\|\nu_{c}\|\right)}^{2}+\lambda (1-T_{c})max{\left(0,\|v_{c}\|-m^{-}\right)}^{2} \end{array}$$ (5)

$$\begin{array}{c} L_{r}={\left(x_{r}-x\right)}^{2},\;L=L_{c}+6*10^{-4}L_{r} \end{array}$$ (6)

where *c* represents the classification category, and *T*_*c*_ represents the indicator function of the classification. If there is a facial expression of the category *c*, then *T*_*c*_ = 1. *m*^+^ represents the upper limit and *m*^−^ represents the lower limit. *x* and *x*_*r*_ denote original image and reconstructed image respectively.

### 3.2 TPN-based expression recognition module (TPN-ERM)

Generally, various facial expressions usually hold different visual tempos. However, there are always higher similarities in some expressions(Fear and Surprise), and the key to distinguish them is their visual tempos. To this end, we propose to use TPN-based expression recognition module (TPN-ERM) to model the variances in visual tempos of different facial expressions precisely to further optimize the performance of expressions recognition system.

#### 3.2.1 Collection of hierarchical features

TPN is built upon a set of M hierarchical features that have increasing temporal receptive fields from bottom to top, and we employ multi-depth pyramid to collect these features from a backbone network, which is defined as:

$$\begin{array}{c} F=\{F_{1},F_{2},\ldots ,F_{M}\} \end{array}$$ (7)

Where *F*_*i*_ represents i-th feature, *F* is a set of *M* hierarchical features, and the size of each feature *F*_*i*_ is:

$$\begin{array}{c} sizes=\{C_{1}\times T_{1}\times W_{1}\times H_{1},\ldots ,C_{M}\times T_{M}\times W_{M}\times H_{M}\} \end{array}$$ (8)

Where *H*, *W*, and *C* represent height, width and numbers of channels of each frame, repectively. *T* is the number of frames. The size usually satisfies the following formula:

$$\begin{array}{c} \left\{C_{i1}\geq C_{i2},W_{i1}\geq W_{i2},H_{i1}\geq H_{i2};i_{i1}\leq i_{i2}\right\} \end{array}$$ (9)

#### 3.2.2 Spatial and temporal semantic modulation

Multi-depth pyramids can well integrate features of different scales, but there is the problem of unaligned spatial semantics. To solve this problem, spatial semantic modulation is applied to TPN. For each feature (top-level features are not included), a series of convolutions with a specific stride are applied, so that the spatial shape and receptive field of these features match the top-level feature. Therefore, the overall loss function of backbone network of TPN is:

$$\begin{array}{c} L_{total}=L_{CE,o}+\sum_{i=1}^{M-1}\lambda_{i}L_{CE,i} \end{array}$$ (10)

where *L*_*CE*,*o*_ is the cross entropy loss, *L*_*CE*,*i*_ is the loss of the i-th auxiliary classifier. {λ_*i*_} is the balance factor. After spatial semantic modulation, features have aligned shapes and consistent semantics in spatial dimensions.

Meanwhile, we also use temporal rate modulation to calibrate in time dimension. In order to improve the flexibility of TPN, we use a set of hyper-parameters ${\left\{\alpha_{i}\right\}}_{i=1}^{M}$ for temporal rate modulation. Specifically, *α* means that the parameter subnet will be used after performing spatial semantic modulation, and the updated feature will be temporarily down-sampled at the i-level using *α*_*i*_ as a factor. The use of such hyper-parameters allows us to better control the differences of features on the temporal scale, so that we can perform feature aggregation more effectively. In the following section, we will call *F*_*i*_ whose size is *C*_*i*_ × *T*_*i*_ × *W*_*i*_ × *H*_*i*_ the i-feature after performing spatial semantic modulation and temporal rate modulation.

#### 3.2.3 Adaptive weighted fusion

After the hierarchical features are collected and preprocessed, the next step is to aggregate these features effectively. Assuming that the aggregated feature at i-th level are $F_{i}^{\prime }$, there are three basic ways:

$$\begin{array}{c} IsolationFlow:F_{i}^{\prime }=F_{i} \end{array}$$ (11)

$$\begin{array}{c} Bottom-upFlow:F_{i}^{\prime }=F_{i}^{\prime }⊕g(F_{i},T_{i}/T_{i-1}) \end{array}$$ (12)

$$\begin{array}{c} Top-downFlow:F_{i}^{\prime }=F_{i}^{\prime }⊕g(F_{i},T_{i}/T_{i+1}) \end{array}$$ (13)

Where ⊕ means element-wise addition. *g*(*F*, *δ*) is applied along the temporal dimension, where *F* represents the feature.

Besides the top-down flow and bottom-up flow, we could also combine them to achieve two other ways, namely Cascade Flow and Parallel Flow. While applying a bottom-up flow after a top-down flow will form the cascade flow, applying them simultaneously will lead to the parallel flow.

**Fig 6:**
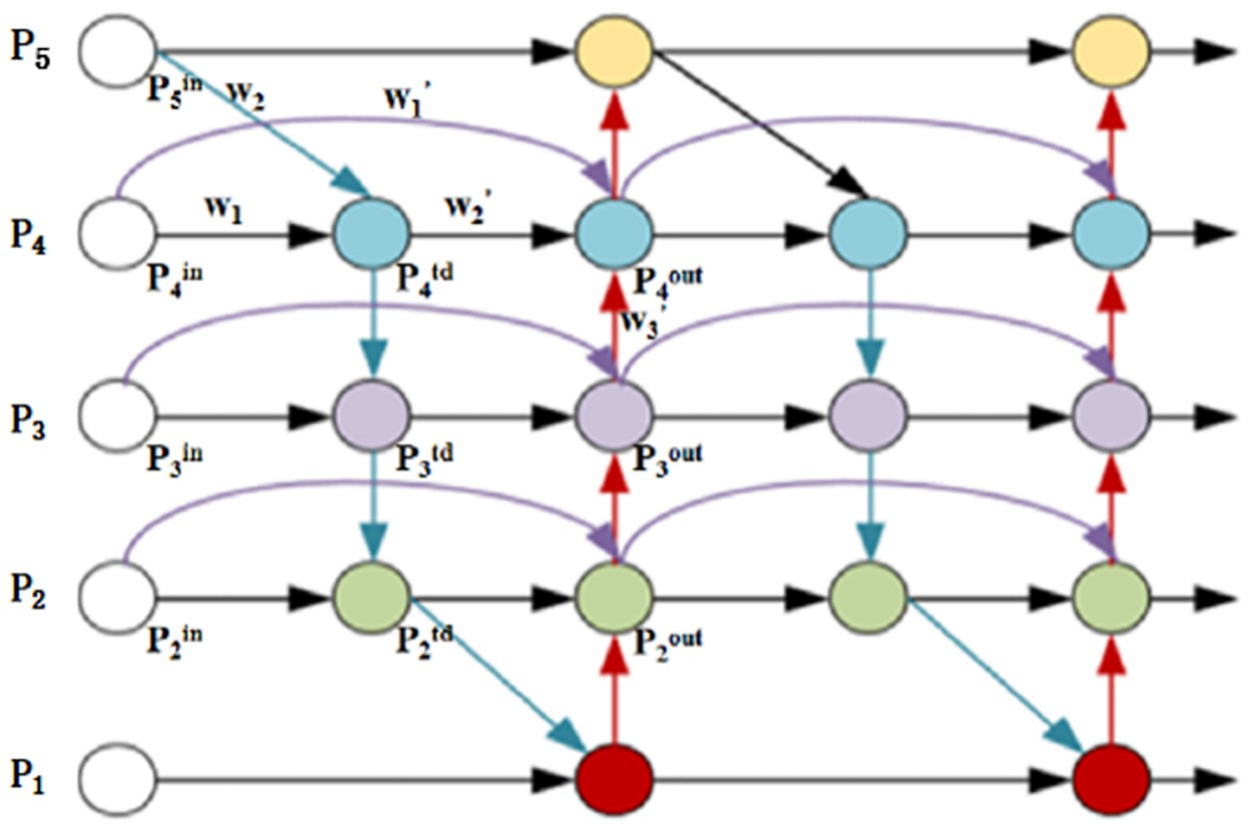
Adaptive weighted fusion. Similar to PANet, but it deletes the nodes with only one in-degree in PANet to eliminate some redundant calculations. Secondly, we added some new connections called cross-node connections shown as the three purple connection curves. The first feature node of the same layer (the white node) in these connections is connected to the output node without top-down feature fusion, while the output nodes participate in bottom-up feature fusion. Finally, inspired by the idea of recursive networks, we integrate top-down with bottom-up flow and add some cross-node connections to enhance fusion of features.

However, TPN does not employ the correlation of features at different levels. Specifically, when these features are aggregated, each of them made a different contribution to the aggregated features. Therefore, based on the FPN [28] and PANet [29], we introduce an adaptive weighted fusion method which can learn parameters to improve the ways of feature aggregation. The top-down and bottom-up flow are integrated and added behind each convolution layer, which can enhance the information fusion. The specific structure is shown in the Fig 6.

Regarding the computational process of feature fusion, the previous pyramid attention network used global self-attention mechanism, but it dose not employ the contribution of input features to the output features is different. Therefore, we apply a weight to each input feature so that the network can learn the importance of each input feature. Two weighted fusion methods are considered:

$$\begin{array}{c} Softmax-based-fusion:O=\sum_{i}\frac{e^{w_{i}}}{\sum_{j}e^{w_{j}}}*I_{i} \end{array}$$ (14)

where *w*_*i*_ is a learnable weight that can be updated during network training, and *I*_*i*_ represents the input weight of i-th layer. The softmax function normalizes the weights to a probability with a range of 0 to 1, which indicates the importance of each input feature. But it will result in more hardware consumption, to minimize additional computational costs, we further propose an efficient fusion method.

$$\begin{array}{c} Fast-standardized-fusion:O=\sum_{i}\frac{w_{i}}{\varepsilon \sum_{j}e^{w_{j}}}*I_{i} \end{array}$$ (15)

Applying the ReLU function behind each *w*_*i*_ can ensure *w*_*i*_ > 0. Fast standardized fusion also makes the value of each weight range between 0 and 1, but it is more efficient than the softmax-based fusion. We take the fourth layer P4 in Fig 6 as an example to describe our adaptive weighted fusion method that uses fast standardized fusion:

$$\begin{array}{c} P_{4}^{td}=Conv(\frac{w_{1}*P_{4}^{in}+w_{2}*Resize\left(P_{5}^{in}\right)}{w_{1}+w_{2}+\varepsilon }) \end{array}$$ (16)

$$\begin{array}{c} P_{4}^{out}=Conv(\frac{w_{1}^{\prime }*P_{4}^{in}+w_{2}^{\prime }*P_{4}^{td}+w_{3}^{\prime }*Resize\left(P_{3}^{out}\right)}{w_{1}^{\prime }+w_{2}^{\prime }+w_{3}^{\prime }+\varepsilon }) \end{array}$$ (17)

among them $P_{4}^{td}$ is the intermediate feature in the top-down path, and $P_{4}^{out}$ is the output feature in the bottom-up path. All other features are constructed in the similar way.

## 4 Experiments

In this section, we first describe two public datasets, and then illustrate the specific experimental details and evaluation metrics. The experiment results demonstrate the effectiveness of our 3D-CapsNet and TPN-ERM. Moreover, we compare our integrated model to current effective models on public datasets. Finally, the ablation studies show the effectiveness of each module in our proposed model.

### 4.1 Datasets

#### 4.1.1 CK+

The Extended Cohn-Kanade (CK+) database [34] collects facial expressions of 123 subjects by videos, and a total of 593 facial expression video sequences. The subjects are young people between ages of 18 and 30. The data sequence vary in duration from 10 to 60 frames. Seven basic emotion classes (anger, contempt, disgust, fear, happiness, sadness, and surprise) are marked in these videos, and the annotation work is based on the Facial Action Coding System (FACS). Since CK+ is not clearly divided into the training set, validation set and test set, the algorithms evaluated on this database are not same. Each image sequence changes from the onset (the neutral frame) to the peak (the expressive frame). Moreover, the X-Y coordinates of 68 facial landmark points were given for each image in the database. The landmark points of key frames within each video sequence were manually labeled, while the remaining frames were automatically aligned using the Active Appearance Model(AAM) fitting algorithm [35].

#### 4.1.2 AFEW

The AFEW database [36] has been used as the official database in the EmotiW since 2013. The AFEW database contains facial expressions collected from different TV and film works which are believed to be closed to real world conditions. The database is comprised of training set, validation set and test set. There are 578 video clips in the training set. The validation and test sets have 383 video clips and 407 video clips, respectively. The video clips are marked with seven expression labels: anger, disgust, fear, happiness, sadness, surprise and neutral. Besides, this database provides original video clips and aligned face sequences. Different from the CK+ database, facial expressions in AFEW are more natural and spontaneous. The variations in illumination, pose and background in image sequences expand the complexity of facial expression analysis.

### 4.2 Experimental settings

#### 4.2.1 Implementation details

We used tensorflow and pytorch frameworks to conduct experiments, and trained the model with a total of 500 iterations. We adopted the ADAM algorithm as the model trainer in the training process, which can utilize hyper-parameters to greatly accelerate the speed of network convergence. In addition, our training parameters on different data sets are slightly different. On the CK+ and AFEW data sets, we set the trainer parameter such as *β*_1_, *β*_2_ and *ε* as 0.9, 0.999 and 10–8. The batch size of each epoch is 16. The value of scaling factor *ρ* is 0.0006 to reconstruction loss in the feature decoding stage.

We used improved TPN for expression auxiliary recognition, which can be integrated into our 3D-CapsNet. We use the inflated 3D-ResNet as the backbone network to ensure good performance on various datasets, and the original ResNet serves as the 2D backbone for contrast. M-level TPN network processes the i-th level features through a series of convolutions with a stride of *M*_*i*_ in spatial semantic modulation and the feature dimension is fixed to 1024. The time rate modulation is realized through the convolution layer and the maximum pooling layer. Finally, the five kinds of information flow mentioned in section 3 aggregate features separately to make a comparison. All aggregated features in TPN will be rescaled by max pooling operation, and their cascade will be fed into the fully connected layer for final prediction.

#### 4.2.2 Evaluation metrics

Multiple performance and evaluation criteria are used to evaluate the performance of proposed model. Following prior work, we adapt Accuracy, Precision(P), Recall(R) and F1 score. The formulas are as follows.

$$\begin{array}{c} Acc=\frac{TP+TN}{TP+TN+FP+FN} \end{array}$$ (18)

$$\begin{array}{c} F_{1}=\frac{2\times P\times R}{P+R} \end{array}$$ (19)

$$\begin{array}{c} P=\frac{TP}{TP+FP},R=\frac{TP}{TP+TN} \end{array}$$ (20)

where TP represents the number of samples correctly predicted as positive class, FP represents the number of samples incorrectly predicted as positive class, TN is the number of samples correctly predicted as negative class, and FN is the number of samples incorrectly predicted as negative class. The same is true for multiple classifications, as long as all other categories that do not belong to the current category are considered as negative cases. Higher values denote better performance for all metrics.

### 4.3 Results of 3D-CapsNet

**Fig 7:**
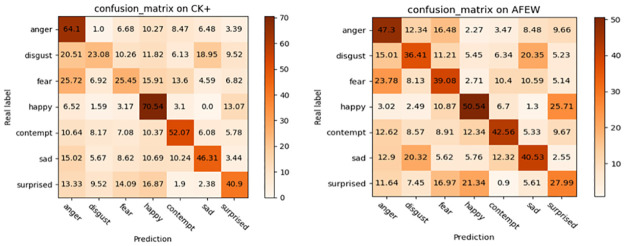
The confusion matrix on CK+(left) and AFEW(right).

First, we drew the confusion matrix for emotion prediction on the CK+ and AFEW data sets, and the results are shown in Fig 7. Through the confusion matrix, we can find that the highest recognition accuracy rate on CK+ is happy, which can reach 70.54%, followed by anger, and the lowest recognition rate is disgust, which only reached 23.08%, because most of disgust expressions are recognized as angry and sad. The expression with higher recognition accuracy rate on AFEW is also happy and angry, reaching 50.54% and 47.3% respectively. The lowest recognition rate is surprise, whose recognition accuracy rate is 27.99%, and quite a few surprise expressions are recognized as happy. To sum up, we can see that the recognition effect of facial expressions with obvious characteristics like happy and angry is far greater than that of contempt and other expressions whose facial features are not obvious, which can also illustrate the necessity of paying attention to the key areas of facial expression during the recognition process.

**Fig 8:**
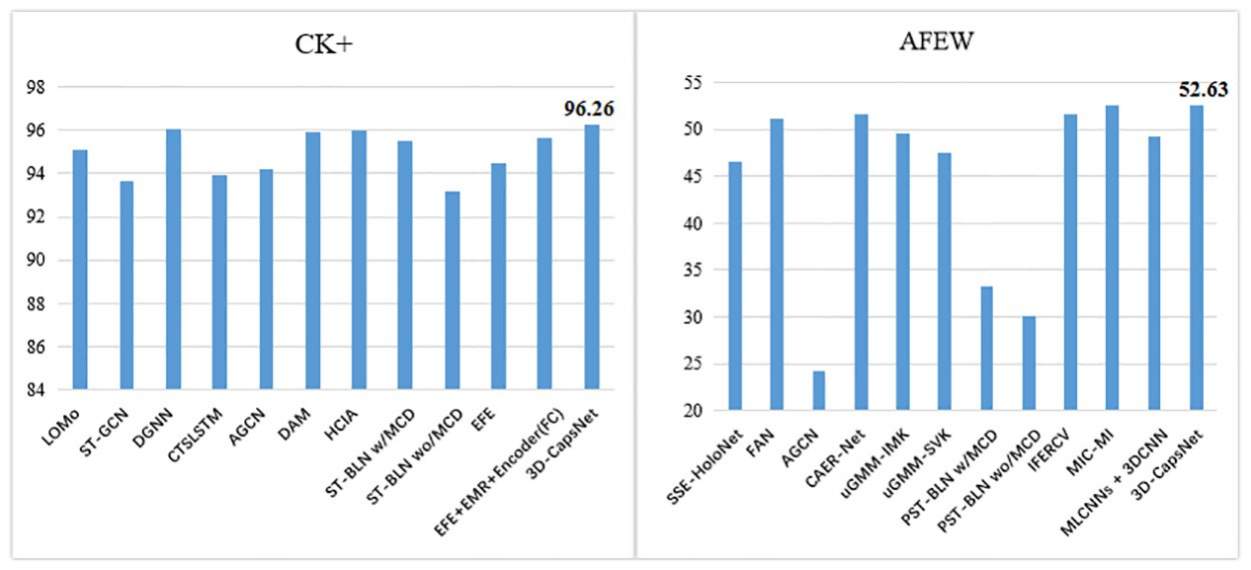
The visualization representation of the experimental results.

We compare our model with other effective methods. These methods mainly enhance their learning ability by capturing global and local features on the corresponding dataset. Considering that the face possesses specific structure, we use dynamic routing between capsules to obtain the relationship between AUs. The capsule network encodes spatial information when calculating the possibility of existence of an object. So it is very suitable for FER. In addition, our network can focus on the facial activity area enhanced by the attention map. In general, the benefits of our model are attributed to two enhancement modules, namely 3D-CNN with AU-perceived attention mechanism and the CapsNet with multiple convolution layers. The performance of the proposed method is compared with both video-based and landmark-based state-of-the-art methods on AFEW and CK+ datasets in Table 2. The visualization representation of the experimental results is shown in Fig 8. Some methods such as [45, 50] use videos or image sequences as the main data stream for training the model while some methods such as [37, 48] utilize local facial landmark data to highlight the most important parts of the facial images and improve the performance. the accuracy of our proposed method on CK+ and AFEW reached 96.26% and 52.63%, respectively, which is better than most methods.

**Table 2:** The performance (%) comparison of 3D-CapsNet with state-of-the-art methods on CK+ and AFEW.

CK+			AFEW		
Methods	Data type	Acc(%)	Methods	Data type	Acc(%)
LOMo [37]	Landmark	95.10	SSE-HoloNet [38]	Video	46.47
ST-GCN [39]	Landmark	93.64	FAN [40]	Video	51.18
DGNN [41]	Landmark	96.02	AGCN [42]	Landmark	24.21
CTSLSTM [43]	Landmark	93.90	CAER-Net [44]	Video	51.68
AGCN [42]	Landmark	94.18	uGMM-IMK [45]	Video	49.50
DAM [46]	Video	95.88	uGMM-SVK [45]	Video	47.50
HCIA [47]	Video	96	PST-BLN w/MCD [48]	Landmark	33.33
ST-BLN w/MCD [48]	Landmark	95.47	PST-BLN wo/MCD [48]	Landmark	30.15
ST-BLN wo/MCD [48]	Landmark	93.19	IFERCV [49]	Video	51.62
EFE [50]	Video	94.44	MIC-MI [51]	Video	52.62
EFE+EMR+Encoder(FC) [50]	Video	95.63	MLCNNs+3DCNN [52]	Video	49.3
3D-CapsNet	Video	96.26	3D-CapsNet	Video	52.63

Then we further analyzed time complexity, space complexity and recognition accuracy of proposed 3D-CapsNet module and other state-ofthe-art modules in terms of the number floating point of operations(FLOPs), the number of model parameters (Params) and accuracy(Acc) in Table 3. It is obvious from Table 3 that our method outperforms others on recognition accuracy. FcaNet utilized a frequency attention method that rethinked channel attention using frequency analysis. Spectral and Spatial Attention (SSA) module integrates spectral semantics with spatial locations to address the problems of interaction between spatial action units and the inadequacy of semantic information about spectral expressions. SA-Net separated channels into two equal portions for channel and spatial attention to address the problem of high computational overhead when fusing spatial attention and channel attention together. Compared to these methods, our model uses fewer parameter quantities and achieves better recognition effect. But the parameter quantity and FLOPs of our model is slightly higher than that of CapsNet. This is because the iteration of dynamic routing in the capsule network results in longer computation time and improved 3D-ResNet introduced extra parameters. But experiment results in Table 3 have shown that the performance of our model is far better than traditional capsule networks and other three models although the importation of extra parameters.

**Table 3:** The FLOPs, Params and accuracy comparison of 3D-CapsNet with state-of-the-art methods.

			Accuracy(%)	
Methods	FLOPs/G	Params/M	CK+	AFEW
SSA [53]	-	23.52	93.56	51.33
SA-Net [54]	-	23.58	92.12	49.80
FcaNet [55]	-	26.04	91.50	48.75
CapsNet [56]	325.37	5.37	76.12	32.30
Ours	445.38	6.40	96.26	52.63

**Fig 9:**
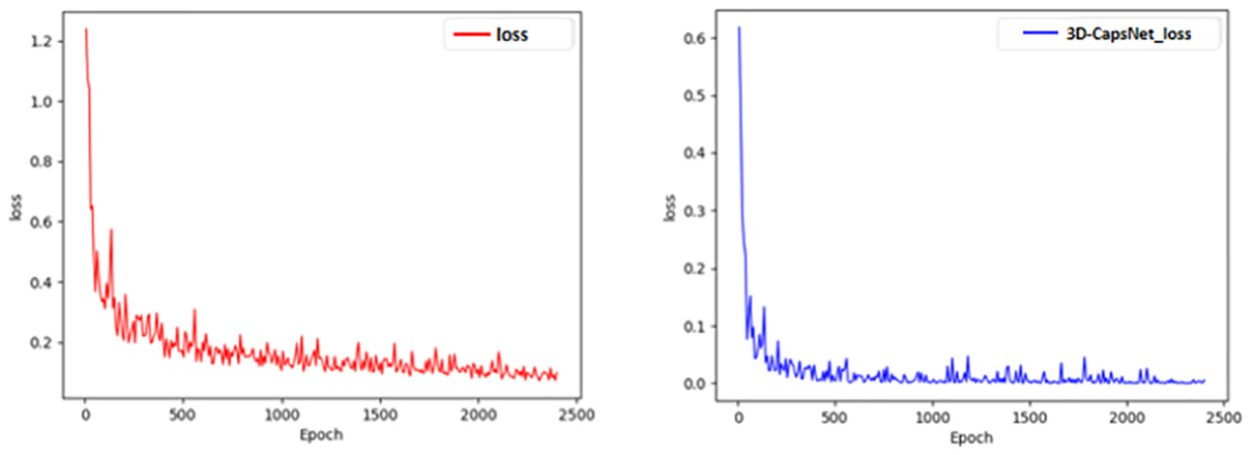
The loss functions of 3D convolution network and 3D-CapsNet.

Through training and experiments with the above methods in section 3, the loss functions of 3D convolution network and our 3D-CapsNet are shown in Fig 9. As can be seen from the figures, the loss of our 3D-CapsNet model is lower than that of single 3D convolution network, and the difference between the predicted value and the true value of the facial expression recognition result gets smaller, which means our model can better optimize the previous facial expression classification model and have better recognition performance.

### 4.4 Impact of TPN-ERM on 3D-CapsNet

In this section, we show the visualization results after applying the improved TPN and compare 3D-CapsNet+TPN-ERM(VT-3DCapsNet) with 3D-CapsNet and other state-of-the-art methods to analyze the impact of TPN-ERM on 3D-CapsNet.

**Fig 10:**
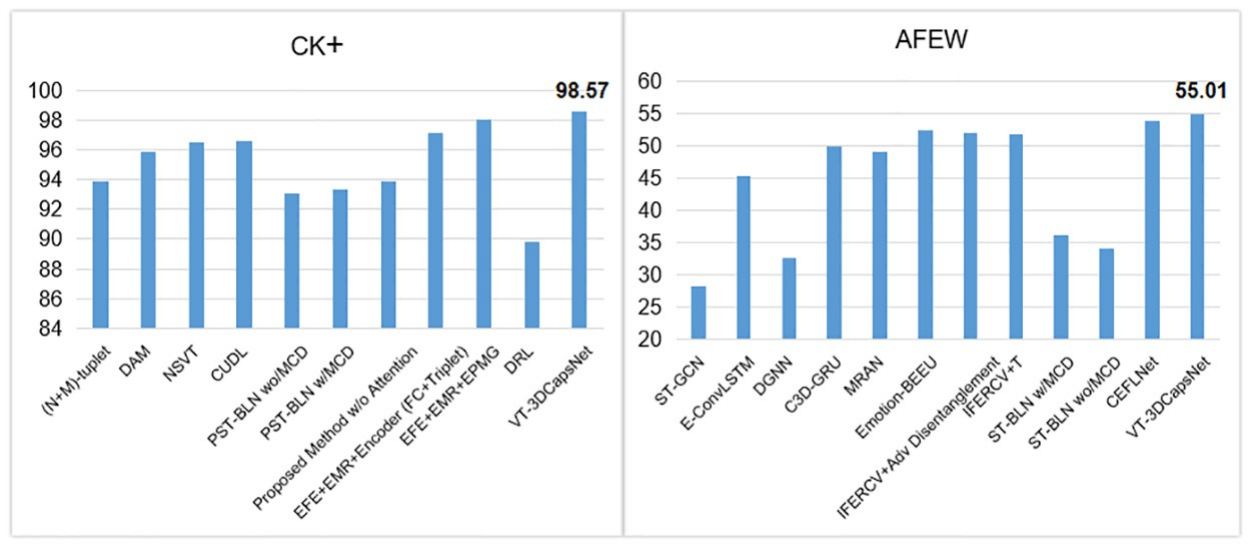
The visualization representation of the experimental results.

We compared the proposed integrated model with other facial expression recognition methods. The results of the comparison experiments are shown in Table 4. The visualization representation of the experimental results is shown in Fig 10.

**Table 4:** The performance (%) comparison of 3D-CapsNet with state-of-the-art methods on CK+ and AFEW.

CK+			AFEW		
Methods	Data type	Acc(%)	Methods	Data type	Acc(%)
(N+M)-tuplet [57]	Landmark	93.90	SSE-HoloNet [38]	Landmark	28.17
DAM [46]	Video	95.88	E-ConvLSTM [58]	Video	45.29
NSVT [59]	Video	96.5	DGNN [41]	Landmark	32.64
CUDL [60]	Video	96.6	C3D-GRU [61]	Video	49.87
PST-BLN wo/MCD [48]	Landmark	93.10	MRAN [62]	Video	49.01
PST-BLN w/MCD [48]	Landmark	93.34	IFERCV+Adv [49]	Video	52.01
Proposed Method w/o Attention [63]	Video	93.84	Emotion-BEEU [64]	Video	52.49
EFE+EMR+Encoder [50]	Video	97.17	IFERCV+*T*^*t*^ [49]	Video	51.86
EFE+EMR+EPMG [50]	Video	98.06	ST-BLN w/MCD [48]	Landmark	36.11
DRL [65]	Video	89.8	ST-BLN wo/MCD [48]	Landmark	34.13
			CEFLNet [66]	Video	53.98
VT-3DCapsNet	Video	96.5	VT-3DCapsNet	Video	55.01

It can be known from the in Table 4 that our method has achieved better and more accurate results on the CK+ and AFEW data sets. Especially, our method can achieve the highest accuracy rate of 98.57% on the CK+, which improves the accuracy by 8.77% compared with DRL in Table 4. The effect on the AFEW data set is also better than most other methods, which can reach an accuracy of 55.01%.

Table 5 shows the precision(P), recall(R), and F1 obtained by our model for recognizing various expressions. From the above table, it can be seen that proposed integrated model(VT-3DCapsNet)has the highest recall rate in terms of happy and surprise, which can achieve 99.68% and 99.34% on the CK+ dataset. The lower recall rates are sad and fear, which only reached 92.82% and 93.74% on CK+, respectively. From the perspective of precision, the precision of happy is the highest, reaching 100% on CK+, while the precision of fear is low, which only achieved 92% on CK+ and 30% on AFEW. F1 represents the model’s ability to recognize various facial expressions. The most prominent F1 values in Table 5 are happy and surprise, which reached 99.8% and 99.1% on CK+, and they also reached optimal values of 66% and 71% on AFEW. The experiment results indicate that our model has the strongest recognition ability for happy and surprise.

**Table 5:** The precision, recall, and F1 obtained by our model for recognizing various expressions.

	AFEW			CK+		
Label	P(%)	R(%)	F1(%)	P(%)	R(%)	F1(%)
anger	37.13	41.92	39.15	95.17	94.32	95.82
disgust	49.43	44.76	47.07	94.57	96.14	95.17
fear	30.00	31.33	31.85	92.00	93.74	93.22
happy	63.21	70.16	66.00	100	99.68	99.80
contempt	46.72	56.25	50.17	97.43	95.61	96.68
sad	33.18	39.34	36.27	95.81	92.82	94.41
surprise	56.29	99.34	71.00	99.70	99.34	99.10

### 4.5 Ablation experiment

Both 3D-CapsNet for feature representations learning and TPN-ERM for optimizing the performance of expressions recognition system gain improvements on FER. We conducted a quantitative evaluation of these two models in order to better understand our method. For a more detailed analysis of the FER results, we also explored how different network components affect the performance of 3D-CapsNet and TPN-ERM.

#### 4.5.1 Evaluation of 3D-CapsNet on AFEW and CK+

In order to prove the effectiveness of the 3D-CapsNet with AU-perceived attention mechanism, we conducted ablation experiments on the AFEW dataset. We compare our model with VGG16 used as basic backbone network for feature extraction. The experimental results are shown in Table 6. It can be seen from the Table 6 that using 3D convolution is better than using VGG16 in processing video tasks. When 3D convolution with AU-perceived attention is used for feature extraction, the model improves the accuracy by 2.1% compared with using VGG16 singly and is also better than the accuracy of the 3D convolution without the attention mechanism. When adding the capsule network, our 3D-CapsNet improves the accuracy by 7% compared to the VGG16 and is slightly better than the model that uses 3D convolution and attention mechanism but does not integrate with the capsule network.

**Table 6:** The ablation study result on AFEW.

3D convolution	capsule neural network	AU perceptual attention	Acc
×	×	×	45.6
√	×	×	46.8
√	×	√	47.7
×	√	×	32.3
√	√	×	48.2
√	√	√	**52.6**

After that, we used different models as contrasts on CK+ to evaluate the influence of the components of 3D-CapsNet and the results are shown in the following Table 7. It can be seen from the table that when the capsule network does not contain multi-layer convolution and attention mechanism is also not used, the recognition accuracy is 76.12% and is relatively low. VGG16 has a deep convolution structure and does not have the dynamic routing mechanism of the capsule network and attention mechanism, which achieves an accuracy of 78.14%. The AVGGNet network contains both deep convolution and attention mechanism and further improve the accuracy to 79.29%. RCCnet contains deep convolution and capsule, and the accuracy rate is increased to 81.12%. Our model contains the attention mechanism of capsule, deep convolution and attention with AU perception and can achieve the highest accuracy rate of 96.26%, thus proving the effectiveness of our method.

**Table 7:** The ablation study result on CK+.

Method	capsule dynamic routing	deep convolution	attention mechanism	Acc
VGG16	×	√	×	78.14
CapsNet	√	×	×	76.12
AVGGNet	×	√	√	79.29
RCCnet	√	√	×	81.12
Ours	√	√	√	**96.26**

**Fig 11:**
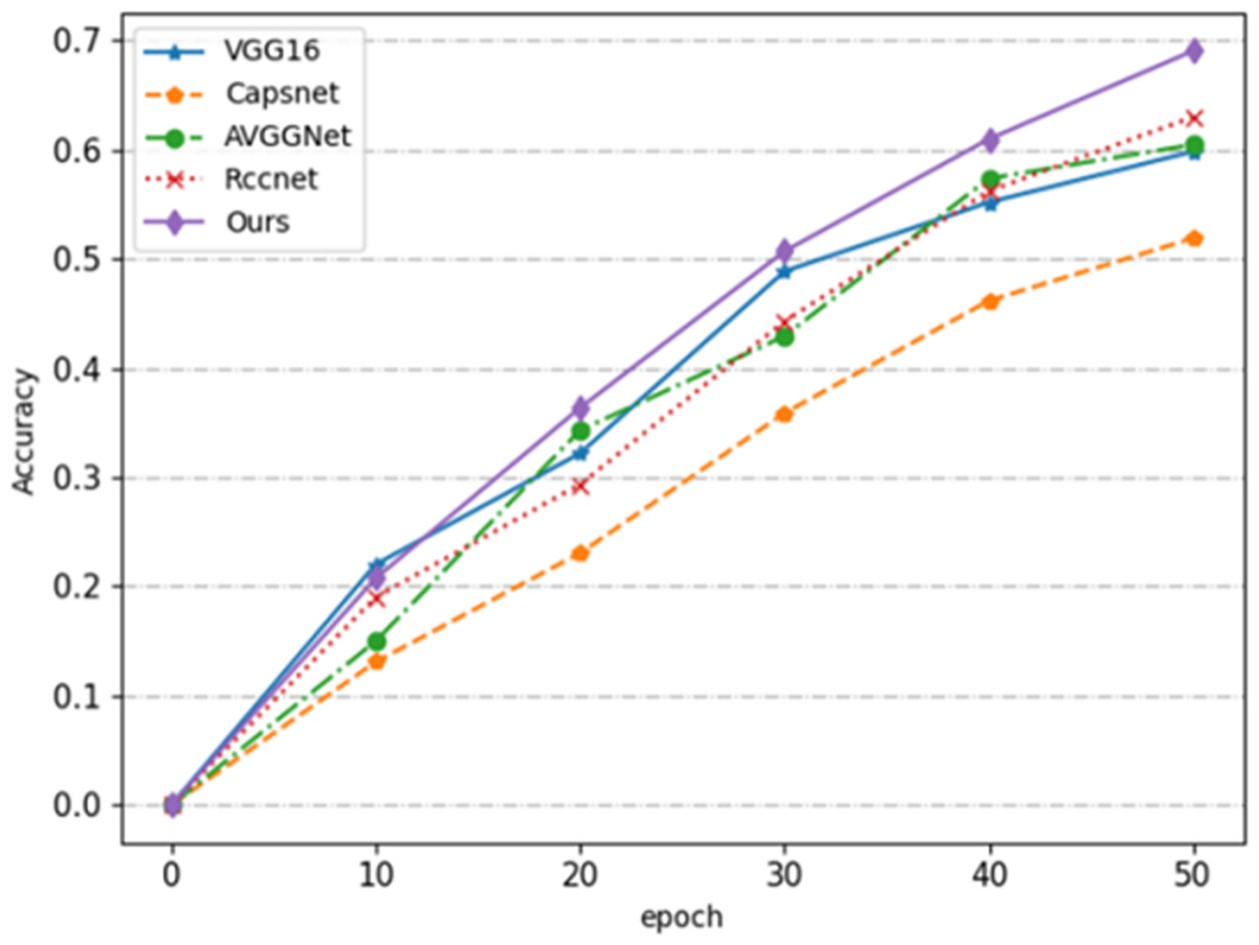
Comparison of the training process of each model.

To illustrate the superiority of our 3D-CapsNet more intuitively, we provide the accuracy curves of our method and other methods on the CK+ data set. As can be seen from the Fig 11, the accuracy of our model in the training process has always been higher than other traditional methods.

#### 4.5.2 Evaluation of TPN-ERM on AFEW and CK+

**Fig 12:**
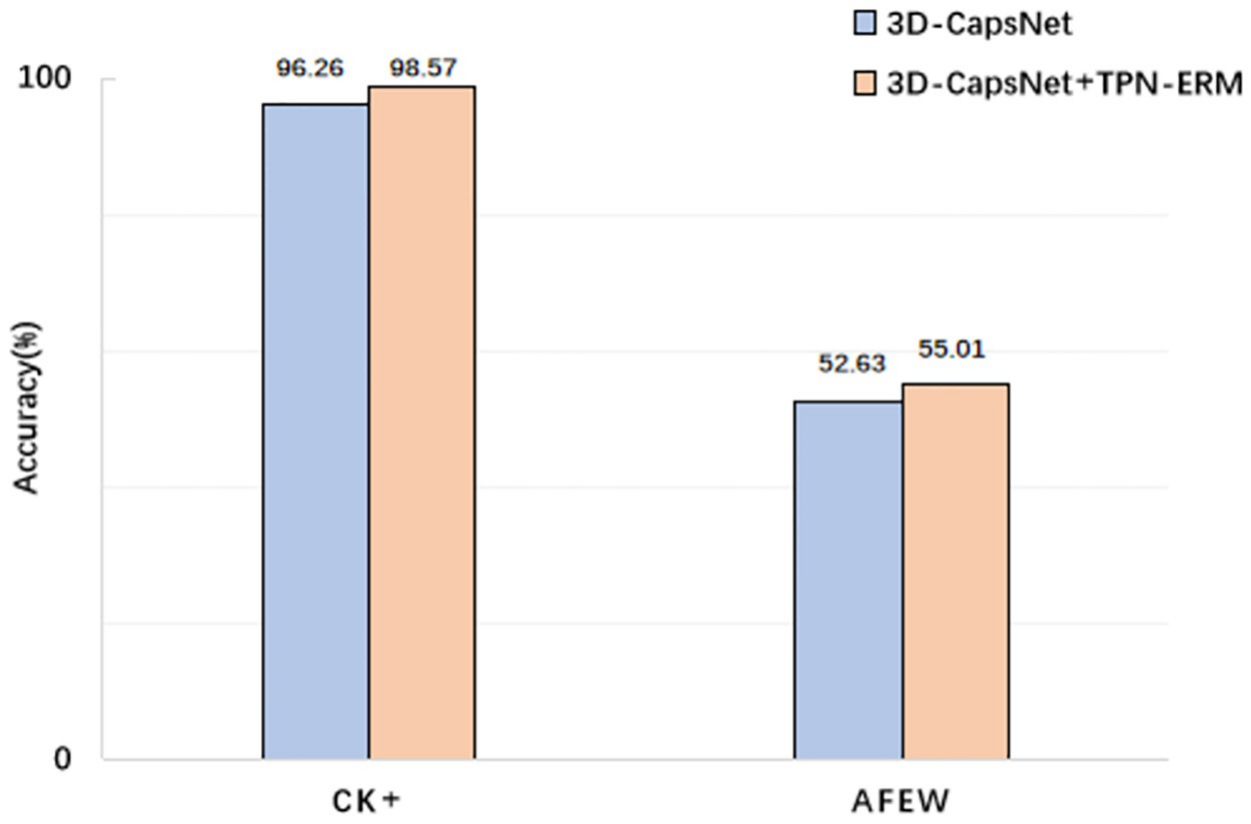
The result of using and not using improved TPN on CK+ and AFEW.

To prove the effectiveness and flexibility of the TPN-ERM we used, we conduct ablation experiments on the CK+ and AFEW datasets. The experimental results are shown in the following Fig 12.

Among them, the purple histogram is the result of only using the 3D-CapsNet model, and the yellow histogram is the result of 3D-CapsNet integrated with improved TPN. It can be seen from the figure that the using the improved TPN can improve the recognition accuracy of the model, which improves the accuracy of 2.31% and 2.38% on the CK+ dataset and AFEW dataset, respectively. These prove that using TPN module is feasible and effective for improving the accuracy of facial expression recognition.

**Fig 13:**
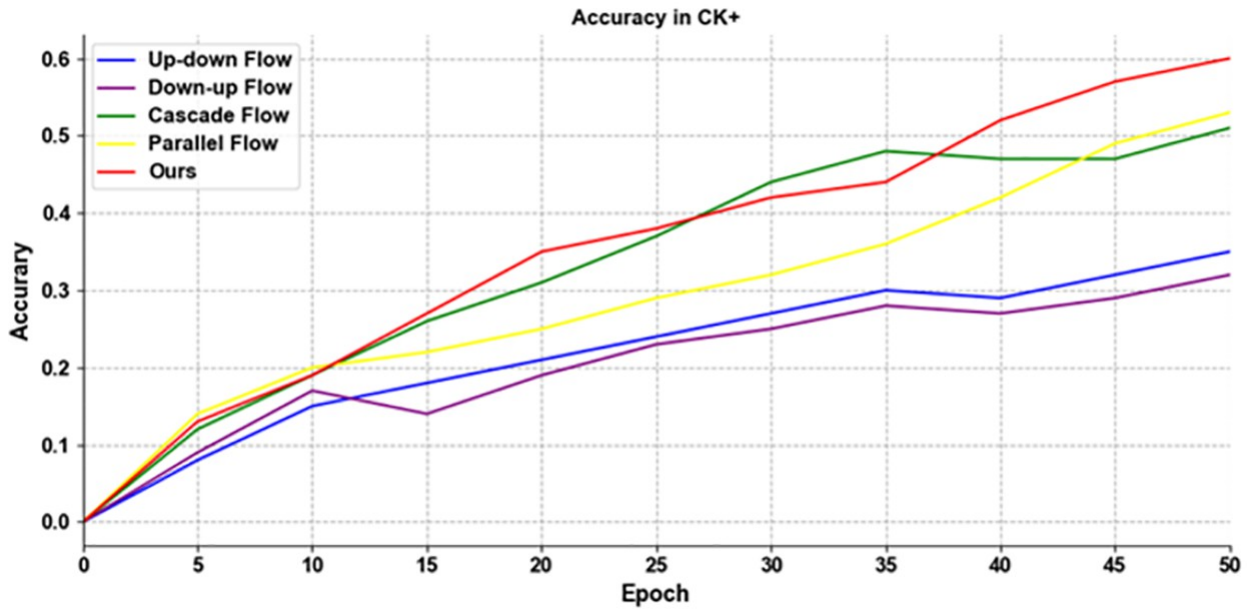
The results of using different information flow methods for TPN training on CK+.

There are four methods of information fusion in the original TPN structure, but we use a novel feature fusion method, namely adaptive weighted fusion. In order to prove that our improvement to TPN are effective, we give the detailed graphs of the accuracy of the first 50 rounds of training on the two data sets in Fig 13.

**Fig 14:**
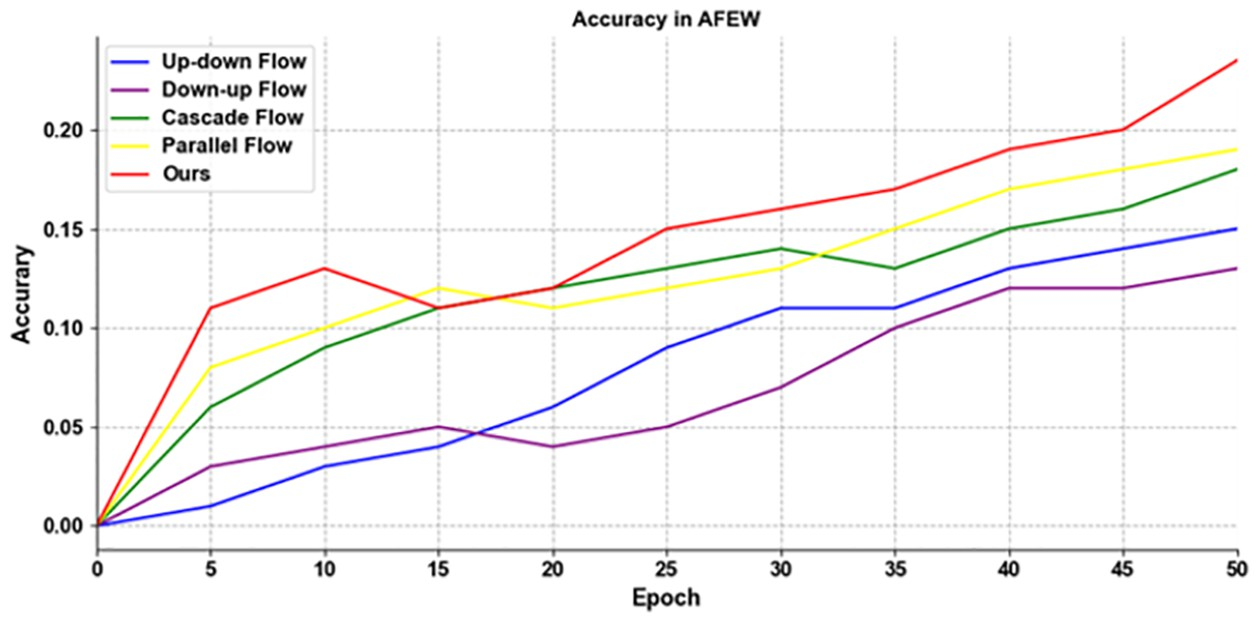
The results of using different information flow methods for TPN training on AFEW.

As can be seen from the Fig 14, our improved TPN with adaptive weighted fusion is more accurate than other information flow fusion methods in the first 50 rounds of training, which proves that using TPN with adaptive weighted fusion is effective.

There are two main calculation methods in our adaptive weighted fusion, namely softmax-based fusion and fast standardized fusion. But the additional softmax will cause more hardware consumption. Therefore, to prove that our fast standardized fusion can process faster, we select a piece of video on the CK+ and compare the processing speed of two calculation methods.

**Fig 15:**
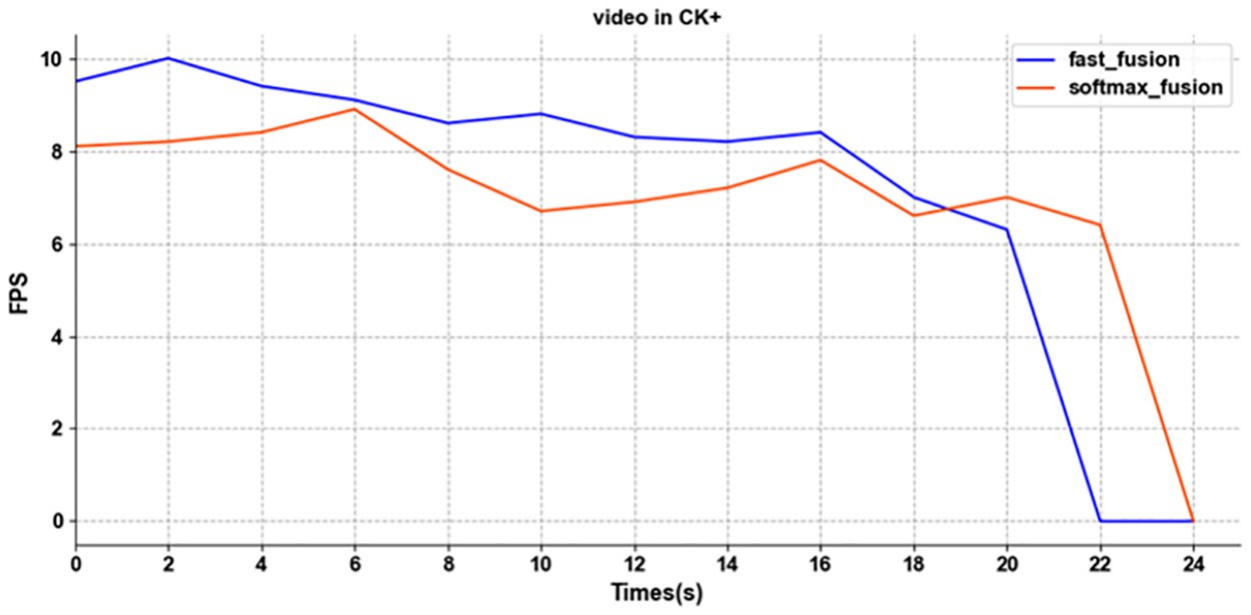
The processing speed result of softmax-based fusion and fast standardized fusion.

The processing speed of faster standardized fusion is significantly faster than that of softmax-based fusion in Fig 15. When the average velocity of processing video is equal, the fast standardized fusion is 2 seconds faster than softmax-based fusion and obtains a better level.

### 4.6 Discussion

In the experimental section of 3D-CapsNet, we aimed to prove that the improved capsule network that applyed improved 3D-ResNet architecture achieved the goal of expressing deeper hierarchical spatiotemporal features and handle complex feature relationships. Furthermore, the confusion matrices on the CK+ and AFEW datasets, when contrasting expressions with obvious features against those with less obvious features, demonstrate a significant impact of facial key regions on the recognition effectiveness. Comparative evaluations with other state-of-the-art models further underscored the outstanding performance of our proposed model in recognition accuracy.

TPN-ERM calculates facial motion features representing expressions from a considerable number of video frames and uses these features as auxiliary outputs to further enhance the performance of 3D-CapsNet. To analysis of TPN-ERM’s impact on 3D-CapsNet, we conducted relevant experiments for performance comparison and complexity analysis, and the results revealed that revealed that TPN-ERM excelled in restore the detailed information of the original images, capturing variations in video, and significantly improving recognition performance of 3D-CapsNet.

To gain a comprehensive understanding of proposed model, we conducted a series of ablation experiments to show the influence of different model components on recognition performance. Specifically, we combined different components of 3D-CapsNet for ablation experiments and validated significant impact of 3D convolution, AU-perceived attention module and capsule neural network on recognition accuracy. Furthermore, we conducted other ablation experiments to prove the impact of TPN-ERM on 3D-CapsNet, and the results established the feasibility and effectiveness of TPN-ERM, which can significantly elevate recognition accuracy of 3D-CapsNet.

Although our model has demonstrated excellent performance in facial expression recognition, there are still some limitations we need to overcome in the future:

1. The proposed 3D-CapsNet model introduces additional parameters compared to the original capsule network, which increases training time and additional hardware memory costs.
2. There are a large number of “ambiguous phenomenon” in more complex real-world scenarios such as low image resolution, occlusion and ambiguous expressions, which can result in low recognition accuracy. So our model can be further improved and discussed in this respect.

## 5 Conclusion

In this paper, we propose a visual tempos 3D-CapsNet to better learn spatiotemporal feature. We also propose a TPN-based expression recognition module (TPN-ERM) that models the variances in visual tempos of facial expressions actions precisely to further optimize the performance of 3D-CapsNet. Extensive experiments demonstrate that 3D-CapsNet outperforms most state-of-the-art models in terms of the accuracy after adding improved 3D-ResNet architecture that integrated with AU-perceived attention module. It also proves that the feature representation of 3D-CapsNet are more informative after integrating with the TPN-ERM. In the future, we will consider optimizing the routing algorithm of CapsNet to reduce the network parameters. Furthermore, the datasets more complex real-world scenarios(low image resolution, occlusion and ambiguous expressions) we used in the experiments are not sufficient, so the performance of expression recognition in complex scenes may not be satisfactory. The next step is to test the performance of our model on relevant datasets for further improvement.
